# Supplementary material for: In Silico Prediction of Mutant HIV-1 Proteases Cleaving a Target Sequence
Source: PLoS One. 2014 May 5;9(5):e95833. doi: 10.1371/journal.pone.0095833 (PMC4010418; doi:10.1371/journal.pone.0095833)
Supplement: File S1 — Tables S1–S11, Figures S1–S14, a movie showing the three dimensional structure of WT-PR bound to the target peptide, with highlighted the residues that are changed in M24. (PDF) [file pone.0095833.s001.pdf]

Supporting Material for

In silico prediction of mutant HIV-1 proteases cleaving a target sequence

Jan H. Jensen, Martin Willemoës, Jakob R. Winther, Luca De Vico

The video animation of the optimized structure of WT-PR binding the target peptide with highlighted residues can be found at this link: http://youtu.be/NEXKojTw2Bc .

Table S1. Cleavable peptides.

	P4	P3	P2	P1	*	P1'	P2'	P3'	P4'
MA-CA	S	Q	N	Y	*	P	I	V	Q
CA-p2	A	R	V	L	*	A	E	A	M
p2-NC	A	T	I	M	*	M	Q	R	G
NC-p1	R	Q	A	N	*	F	L	G	K
p1-p6	P	G	N	F	*	L	Q	S	R
p6pol-PR	S	F	N	F	*	P	Q	V	T
TF-PR	S	F	N	F	*	P	Q	I	T
PR-RTp51	T	L	N	F	*	P	I	S	P
RTp51-RTp66	A	E	T	F	*	Y	V	D	G
RTp66-INT	R	K	V	L	*	F	L	D	G
RH-IN	R	K	I	L	*	F	L	D	G

List of the cleavable endogenous peptides considered in this work

Table S2. Extra cleavable peptides.

	P4	P3	P2	P1	*	P1'	P2'	P3'	P4'
K001	T	Q	I	M	*	F	E	T	F
K002	G	Q	V	N	*	Y	E	E	F
K003	P	F	I	F	*	E	E	E	P
K005	D	T	V	L	*	E	E	M	S
K007	A	E	E	L	*	A	E	I	F
K008	S	L	N	L	*	R	E	T	Q
K010	A	E	C	F	*	R	I	F	D
K011	D	Q	I	L	*	I	E	I	C
K012	D	D	L	F	*	F	E	A	D
K013	Y	E	E	F	*	V	Q	M	M
K014	P	I	V	G	*	A	E	T	F
K016	R	E	A	F	*	R	V	F	D
K018	A	Q	T	F	*	Y	V	N	L
K019	P	T	L	L	*	T	E	A	P
K020	S	F	I	G	*	M	E	F	K
K021	D	A	I	N	*	T	E	F	K
K022	Q	I	T	L	*	W	Q	R	P
K023	E	L	E	F	*	P	E	G	G
K029	K	E	L	Y	*	P	L	T	S
K031	S	R	S	L	*	Y	A	S	S
K032	A	E	A	M	*	S	Q	V	T
K034	G	S	H	L	*	V	E	A	L
K035	G	G	V	Y	*	A	T	R	S
K036	F	R	S	G	*	V	E	T	T
K037	V	E	V	A	*	E	E	E	E
K038	L	P	V	N	*	G	E	F	S
K039	E	T	T	A	*	L	V	C	D
K040	H	L	V	E	*	A	L	Y	L
K041	H	Y	G	F	*	P	T	Y	G
K042	D	S	A	D	*	A	E	E	D
K043	G	W	I	L	*	G	E	H	G
K045	Q	A	I	Y	*	L	A	L	Q
K046	E	K	V	Y	*	L	A	W	V
K047	V	E	I	C	*	T	E	M	E
K048	T	Q	D	F	*	W	E	V	Q
K049	L	W	M	G	*	Y	E	L	H
K050	G	D	A	Y	*	F	S	V	P
K051	E	L	E	L	*	A	E	N	R
K052	S	K	D	L	*	I	A	E	I
K053	L	E	V	N	*	I	V	T	D
K054	I	I	V	A	*	C	E	G	N
K056	G	G	N	Y	*	P	V	Q	H
K057	A	R	L	M	*	A	E	A	L
K058	P	F	A	A	*	A	Q	Q	R
K059	P	R	N	F	*	P	V	A	Q
K060	G	L	A	A	*	P	Q	F	S
K061	S	L	N	L	*	P	V	A	K
K063	R	Q	V	L	*	F	L	E	K
K064	Q	M	I	F	*	E	E	H	G
SUB3	Q	I	T	L	*	W	K	R	P
T035	V	E	I	C	*	T	E	M	E
T084	T	Q	D	F	*	W	E	V	Q
T112	G	D	A	Y	*	F	S	V	P
T228	L	W	M	G	*	Y	E	L	H
T300	E	L	E	L	*	A	E	N	R
T322	S	K	D	L	*	I	A	E	I
T480	Q	A	I	Y	*	L	A	L	Q
T491	L	E	V	N	*	I	V	T	D
T529	E	K	V	Y	*	L	A	W	V

List of the cleavable non-endogenous peptides considered in this work

Table S3. Other peptides

	P4	P3	P2	P1	*	P1’	P2’	P3’	P4’
Target	H	L	S	F	*	M	A	I	P
NC-p1^{A431V}	R	Q	V	N	*	F	L	G	K
NC-p1^{K436R}	R	Q	A	N	*	F	L	G	R
poli-Ala	A	A	A	A	*	A	A	A	A
poli-Arg	R	R	R	R	*	R	R	R	R

List of other peptides considered in this work

Table S4. Non-cleavable peptides

	P4	P3	P2	P1	*	P1'	P2'	P3'	P4'
NBP1	V	N	C	A	*	K	K	I	V
NBP2	W	R	N	R	*	C	K	G	T
NBP3	M	M	K	S	*	R	N	L	T
NBP4	L	A	A	A	*	M	K	R	H
NBP5	T	T	Q	A	*	N	K	H	I
T015	G	M	D	G	*	P	K	V	K
T031	I	K	A	L	*	V	E	I	C
T033	A	L	V	E	*	I	C	T	E
T037	I	C	T	E	*	M	E	K	E
T039	T	E	M	E	*	K	E	G	K
T080	L	N	K	R	*	T	Q	D	F
T082	K	R	T	Q	*	D	F	W	E
T086	D	F	W	E	*	V	Q	L	G
T088	W	E	V	Q	*	L	G	I	P
T108	V	L	D	V	*	G	D	A	Y
T110	D	V	G	D	*	A	Y	F	S
T114	A	Y	F	S	*	V	P	L	D
T116	F	S	V	P	*	L	D	E	D
T224	E	P	P	F	*	L	W	M	G
T226	P	F	L	W	*	M	G	Y	E
T230	M	G	Y	E	*	L	H	P	D
T232	Y	E	L	H	*	P	D	K	W
T296	T	E	E	A	*	E	L	E	L
T298	E	A	E	L	*	E	L	A	E
T302	E	L	A	E	*	N	R	E	I
T304	A	E	N	R	*	E	I	L	K
T318	Y	Y	D	P	*	S	K	D	L
T320	D	P	S	K	*	D	L	I	A
T324	D	L	I	A	*	E	I	Q	K
T326	I	A	E	I	*	Q	K	Q	G
T441	Y	V	D	G	*	A	A	N	R
T476	K	T	E	L	*	Q	A	I	Y
T478	E	L	Q	A	*	I	Y	L	A
T482	I	Y	L	A	*	L	Q	D	S
T484	L	A	L	Q	*	D	S	G	L
T487	Q	D	S	G	*	L	E	V	N
T489	S	G	L	E	*	V	N	I	V
T493	V	N	I	V	*	T	D	S	Q
T495	I	V	T	D	*	S	Q	Y	A
T525	L	I	K	K	*	E	K	L	A
T527	K	K	E	K	*	V	Y	L	A
T531	V	Y	L	A	*	W	V	P	A
T533	L	A	W	V	*	P	A	H	K

List of non-cleavable peptides considered in this work

Table S5. Computed binding energies of WT-PR and non-endogenous cleavable peptides.

Substrate Peptide	FMO (kcal/mol)	RosettaDock (kT)	Substrate Peptide	FMO (kcal/mol)	RosettaDock (kT)
K001	-22	-2	K043	-67	-5
K002	-33	-4	K045	-55	-5
K003	-16	-8	K046	-39	-4
K005	-24	-4	K047	-79	-5
K007	-27	-1	K048	-86	-9
K008	-40	-2	K049	-40	-5
K010	-55	-4	K050	-36	-7
K011	-42	-3	K051	-31	-2
K012	-22	-7	K052	-4	1
K013	-36	-2	K053	-51	-4
K014	-29	-3	K054	-56	-1
K016	-73	-3	K056	-70	-5
K018	-72	-4	K057	-7	-6
K019	-61	-9	K058	-35	-2
K020	-8	-3	K059	-58	-6
K021	-64	-8	K060	-36	-5
K022	-45	-5	K061	-34	-5
K023	-63	-11	K063	-61	-3
K029	-48	-4	K064	-2	-7
K031	-32	-5	SUB3	-30	-6
K032	-51	-6	T035	-67	-4
K034	-8	-8	T084	-81	-7
K035	-30	-8	T112	-69	-11
K036	-3	-3	T228	-29	-5
K037	-2	-4	T300	-29	-1
K038	-39	-5	T322	-21	1
K039	-10	0	T480	-62	-5
K040	-30	-6	T491	-56	-1
K041	-53	-6	T529	-27	1
K042	-29	-6			

Table S6. Computed binding energies of WT-PR and non-cleavable peptides.

Substrate Peptide	FMO (kcal/mol)	RosettaDock (kT)	Substrate Peptide	FMO (kcal/mol)	RosettaDock (kT)
NBP1	-18	3	T296	43	2
NBP2	-21	3	T298	-54	-2
NBP3	-63	0	T302	-44	4
NBP4	-18	4	T304	1	-2
NBP5	-68	7	T318	23	0
T015	2	-3	T320	23	-2
T031	-29	-6	T324	-18	0
T033	-10	-2	T326	-10	5
T037	-28	-5	T441	-30	-2
T039	-36	-3	T476	-10	1
T080	-45	4	T478	-33	-2
T082	9	-2	T482	-45	-1
T086	-23	0	T484	-24	-2
T088	-42	3	T487	31	-3
T108	-12	-4	T489	-24	-6
T110	-21	-3	T493	-19	-1
T114	-16	-8	T495	-15	1
T116	-10	-2	T525	61	7
T224	48	-1	T527	-9	1
T226	-49	-9	T531	-5	-5
T230	19	-2	T533	-23	-7
T232	-13	-1			

Table S7. Strategy1 suggested mutant PRs.

Mutant ID	Chain A	Chain B	Mutation Scheme	Notes
**M1**	V82R I84V	D30Y V82I	A	
**M2**	V82Y	D30V	B	
**M3**	D30T	D30V V82I	B F	
**M4**	D30Y V82R	D30Y V82R	–	Homodimer of **M1**
**M5**	D30V V82Y	D30V V82Y	–	Homodimer of **M2**
**M6**	D30V	D30V	–	Homodimer of **M3**
**M7**	D30T I84V	D30V V82F	A F	Initial temperature = 9 kT
**M8**		D30V	B F	Initial temperature = 9 kT
**M9**	D30T I47L L76F V82R I84T	D30E V82Y	A	Initial temperature = 6 kT
**M10**	V82Y	D30T I84L	B	Initial temperature = 6 kT
**M11**	D30V V82Y I84V	D30H I47L L76F V82Y	A	Initial temperature = 12 kT
**M12**	V82Y	D30T	B	Initial temperature = 12 kT
**M13**	D30E L76F V82R	D30E L76F V82R	–	Homodimer of **M9**
**M14**	D30T V82Y I84L	D30T V82Y I84L	–	Homodimer of **M10**
**M15**	D30H I47L V82Y	D30H I47L V82Y	–	Homodimer of **M11**
**M16**	D30T V82Y	D30T V82Y	–	Homodimer of **M12**

Different mutant PRs were obtained by small modifications of the mutation algorithm. Inside Strategy1 two different schemes were used when choosing which residues could mutate. In Scheme A all six specificity determining residues were allowed to mutate on both chains. In Scheme B only residues 76 and 82 were set as mutable on Chain A and 30, 47, 48, and 84 on Chain B. In addition, a straight forward variant of the algorithm was tested, as opposed to the step-wise one presented in Table S10. In this variant (Scheme F) the protease was directly 'exposed' to the final target peptide sequence. A K value in the order of 20 was necessary. Other parameters that differ from those specified in the Computational Methods section are also highlighted.

Table S8. FMO computed binding energies of HIV-1 protease WT and Strategy1 mutant PRs.

PR	Peptides Target	poly-Ala	poly-Arg	TF-PR	CA-p2	p2-NC
WT-PR	-9	-15	-41	-62	-52	-72
**M1**	-7	3	28	-41		
**M2**	-13	-9	12	-41		
**M3**	-8	-4	13	-43		
**M4**	-18	-24	17	-52		
**M5**	-30	-14	4	-54	-27	-73
**M6**	-14	2	-1	-14		
**M7**	-7	-2	3	-49		
**M8**	-10	-9	-5	-55		
**M9**	20	17	14	-36		
**M10**	40	11	-3	-43		
**M11**	5	10	45	-44		
**M12**	2	2	16	-43		
**M13**	3	2	-24	-52		
**M14**	3	8	17	-60		
**M15**	-9	2	71	-51		
**M16**	-8	-6	26	-44		

Computed binding energies (kcal/mol) of WT-PR and Strategy1 mutant proteases in complex with target, poly-alanine, poly-arginine, TF-PR, CA-p2 and p2-NC peptides.

Table S9. Residues set as mutable in Strategy 2.

Chain A	Chain B
Arg 8	Arg 8
Ala 28	Leu 23
Asp 29	Ala 28
Asp 30	Asp 29
Val 32	Asp 30
Gly 48	Lys 45
Gly 49	Ile 47
Ile 50	Gly 48
Leu 76	Gly 49
Thr 80	Ile 50
Pro 81	Pro 81
Val 82	Val 82
Ile 84	Ile 84

The residues were selected as those inside a 3 Å radius from the substrate peptide plus the specificity determining residues, if not included, minus the catalytic triad Asp25, Thr26 and Gly27 on both chains. The optimized structure of WT protease in complex with the TF-PR peptide was used as template.

Table S10. Substrate peptide mutation sequence

	P4	P3	P2	P1	*	P1'	P2'	P3'	P4'
Start	Ser	Phe	Asn	Phe	*	Pro	Gln	Ile	Thr
	His	Phe	Asn	Phe	*	Pro	Gln	Ile	Thr
	His	Phe	Asn	Phe	*	Pro	Gln	Ile	Pro
	His	Leu	Asn	Phe	*	Pro	Gln	Ile	Pro
	His	Leu	Asn	Phe	*	Pro	Ala	Ile	Pro
	His	Leu	Ser	Phe	*	Pro	Ala	Ile	Pro
Target	His	Leu	Ser	Phe	*	Met	Ala	Ile	Pro

Step wise sequence of substrate peptides employed in the mutation algorithm. The starting sequence corresponds to the natural substrate TF-PR. This sequence is altered one amino acid at the time towards that of the desired target sequence. The P1 and P3' position were not changed during the sequence.

Table S11. ROC data.

Total							
FMO				Rosetta			
cutoff	True positive	False positive	Distance to (0, 1)	cutoff	True positive	False positive	Distance to (0, 1)
-inf	1.00	1.00	1.00	-inf	1.00	1.00	1.00
-10	0.90	0.67	0.68	4	1.00	0.88	0.88
-15	0.89	0.56	0.57	3	1.00	0.84	0.84
-20	0.87	0.44	0.46	2	1.00	0.77	0.77
-25	0.81	0.30	0.35	1	0.99	0.74	0.74
-30	0.71	0.26	0.38	0	0.94	0.63	0.63
-35	0.61	0.21	0.44	-1	0.93	0.58	0.59
-40	0.53	0.19	0.51	-2	0.84	0.37	0.40
-45	0.47	0.09	0.54	-3	0.74	0.23	0.35
-50	0.44	0.07	0.56	-4	0.61	0.19	0.43
-55	0.39	0.05	0.62	-5	0.50	0.14	0.52
-60	0.30	0.05	0.70	-6	0.27	0.09	0.73
-65	0.17	0.02	0.83	-7	0.20	0.05	0.80
-70	0.09	0.00	0.91	-8	0.13	0.05	0.87
-75	0.04	0.00	0.96	-9	0.06	0.00	0.94
-80	0.03	0.00	0.97	-10	0.04	0.00	0.96
-85	0.01	0.00	0.99	-11	0.01	0.00	0.99
+inf	0.00	0.00	1.00	+inf	0.00	0.00	1.00
Only endogenous							
FMO				Rosetta			
cutoff	True positive	False positive	Distance to (0, 1)	cutoff	True positive	False positive	Distance to (0, 1)
-inf	1.00	1.00	1.00	-inf	1.00	1.00	1.00
-10	1.00	0.67	0.67	4	1.00	0.88	0.88
-15	1.00	0.56	0.56	3	1.00	0.84	0.84
-20	1.00	0.44	0.44	2	1.00	0.77	0.77
-25	1.00	0.30	0.30	1	0.91	0.74	0.75
-30	1.00	0.26	0.26	0	0.91	0.63	0.63
-35	1.00	0.21	0.21	-1	0.91	0.58	0.59
-40	1.00	0.19	0.19	-2	0.91	0.37	0.38
-45	0.91	0.09	0.13	-3	0.91	0.23	0.25
-50	0.82	0.07	0.19	-4	0.73	0.19	0.33
-55	0.73	0.05	0.28	-5	0.73	0.14	0.31
-60	0.64	0.05	0.37	-6	0.36	0.09	0.64
-65	0.27	0.02	0.73	-7	0.36	0.05	0.64
-70	0.09	0.00	0.91	-8	0.27	0.05	0.73
-75	0.00	0.00	1.00	-9	0.18	0.00	0.82
-80	0.00	0.00	1.00	-10	0.09	0.00	0.91
-85	0.00	0.00	1.00	-11	0.09	0.00	0.91
+inf	0.00	0.00	1.00	+inf	0.00	0.00	1.00

Comparison of ROC data for FMO energy re-evaluation and RosettaDock energy function generated binding energies, while considering different cutoff values. The upper part of the table reports the full comparison between known cleavable and non cleavable peptides. In the lower part, data for only the endogenous peptides was used for the cleavable part. True positive data is reported in the graphs as sensitivity, false positive as 1 - specificity. Theoretical values for $\pm$ infinite cutoff have been added.

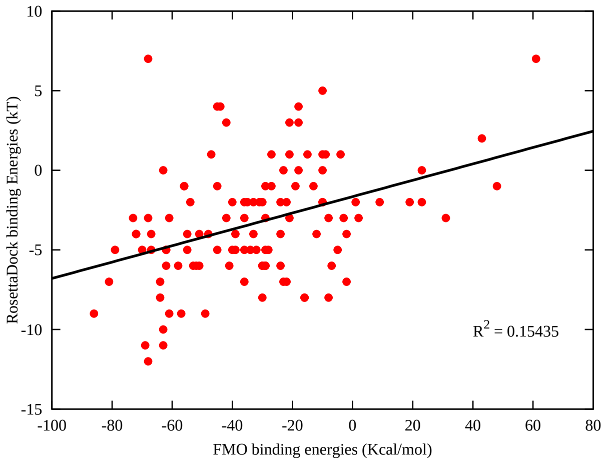

Figure S1. Correlation plot between FMO and RosettaDock computed binding energies. The linear trend line shows no correlation between the data (R^2 = 0.15435).

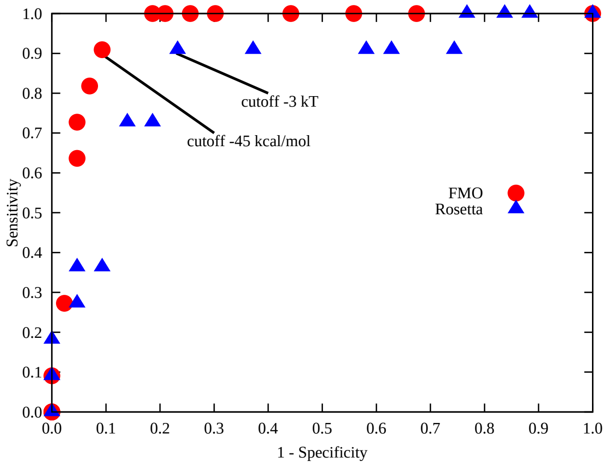

Figure S2. ROC plot comparing different cutoff values for binding energies computed through FMO energy re-evaluation or RosettaDock energy function. The values for each method closest to the theoretical optimum (0,1) are highlighted. The comparison was done using the data of only the endogenous peptides for the cleavable peptides part. The computed area under the ROC curve is 0.96 and 0.84 for FMO and Rosetta, respectively. The raw data is reported in Table S11 in File S1.

Figures S3 - S14 compare the changes between WT-PR and **M24** , residue by residue. In each figure the enzyme is represented as semi-transparent ribbon, the peptide as sticks and the changing residue as ball-and-sticks. The peptide residues numbering is from 2 to 9. Each residue changed by Strategy2 is indicated by a label containing the chain, the residue name and its number.

A28S

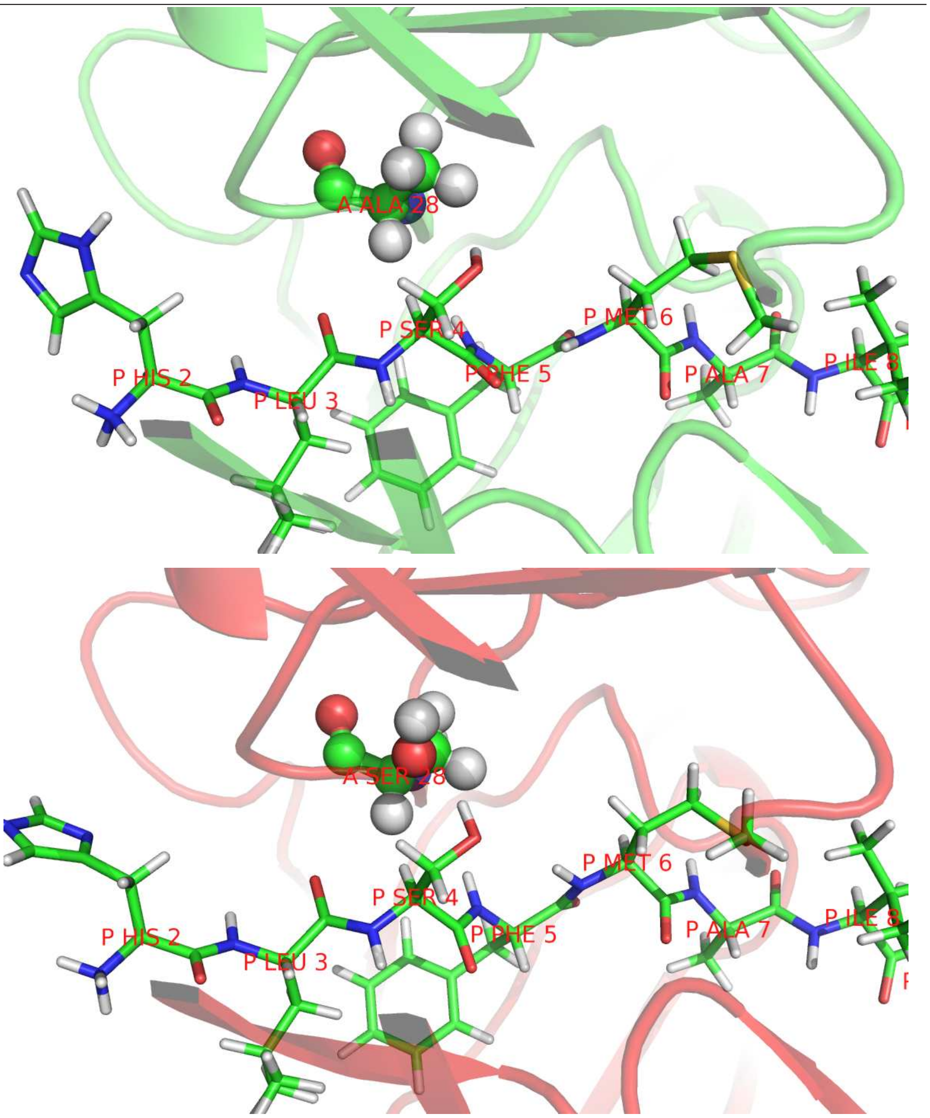

WT

M24

Figure S3. Chain A, residue 28.

D30T

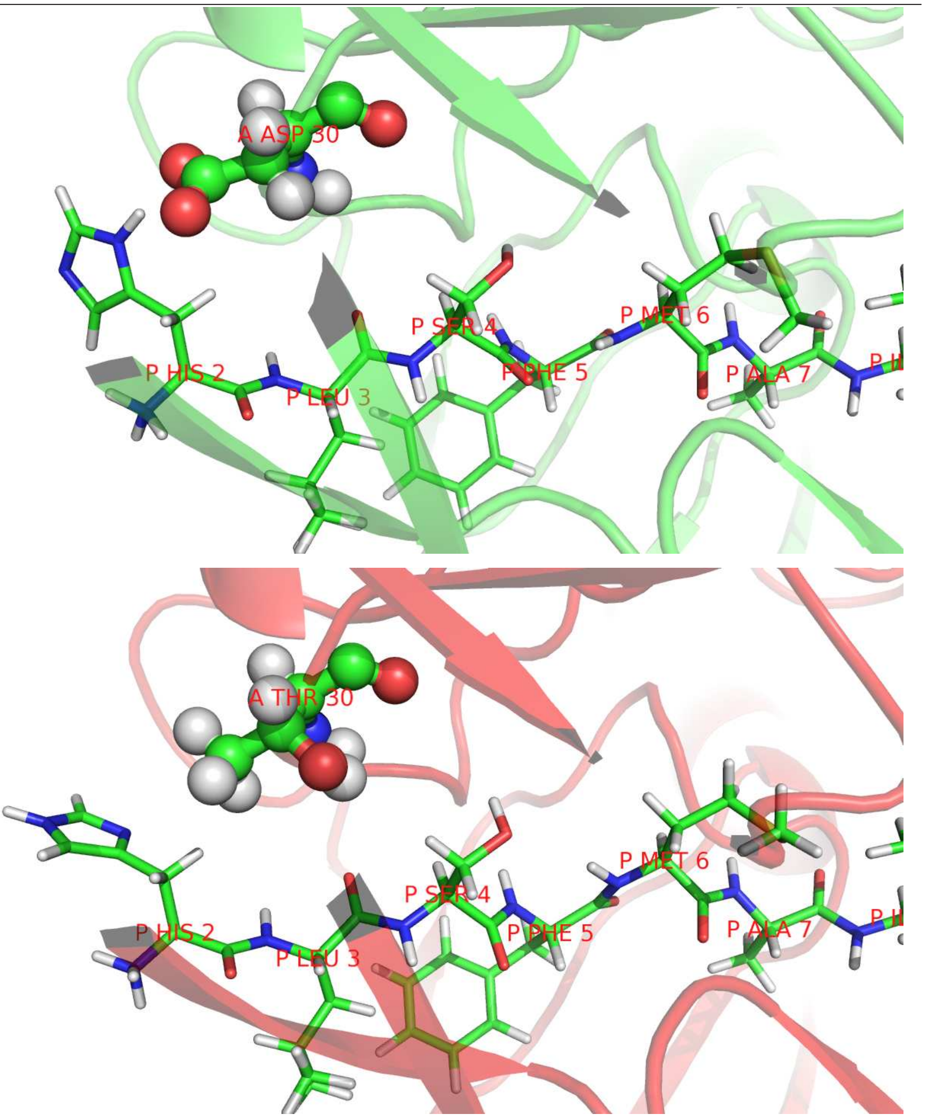

WT

M24

Figure S4. Chain A, residue 30.

K45A

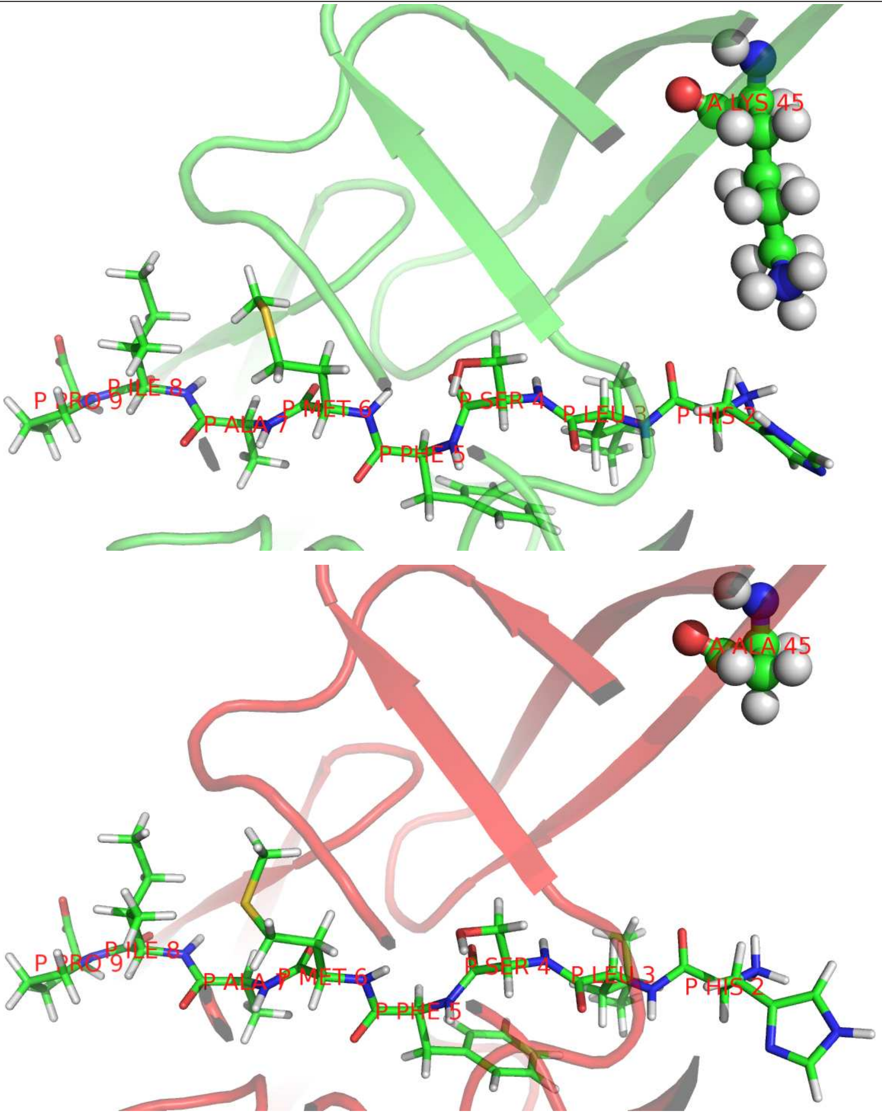

WT

M24

Figure S5. Chain A, residue 45.

I50L

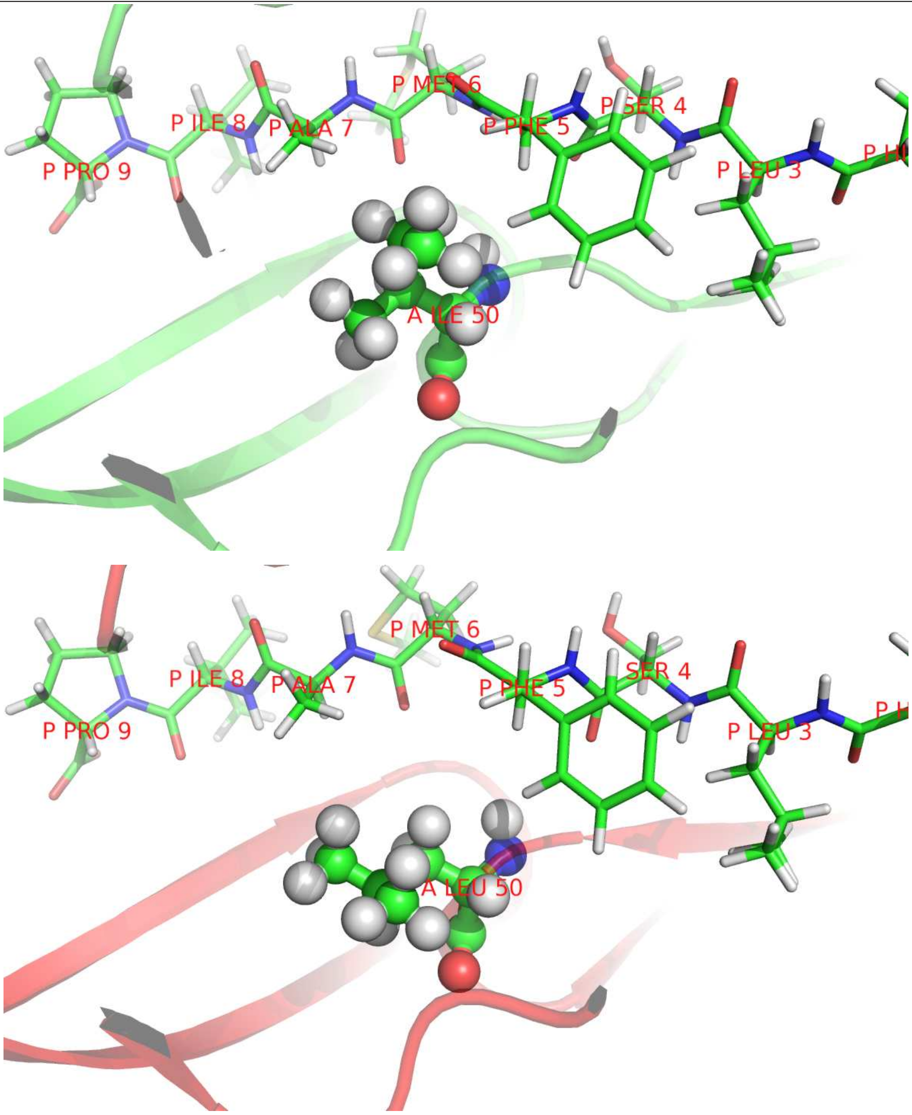

WT

M24

Figure S6. Chain A, residue 50.

P81L

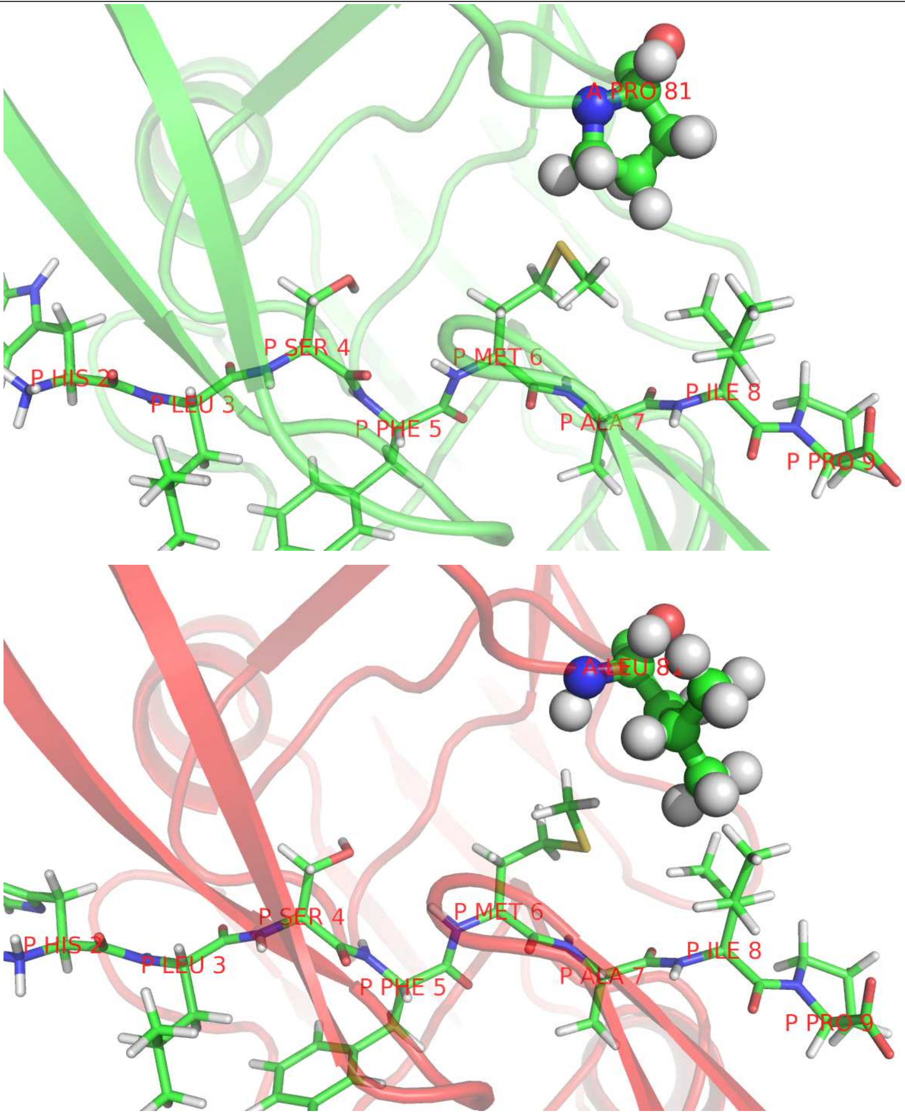

WT

M24

Figure S7. Chain A, residue 81.

V82Y

WT

M24

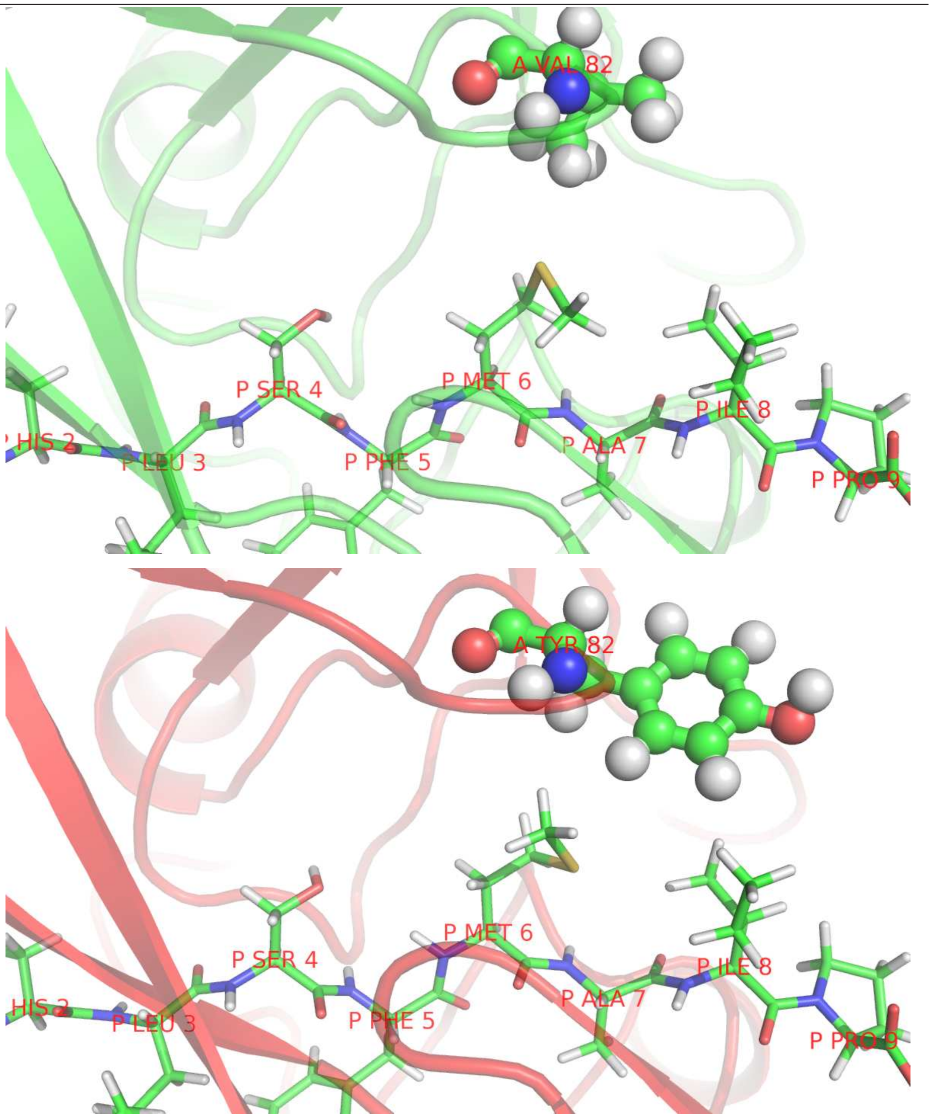

Figure S8. Chain A, residue 82.

A28S

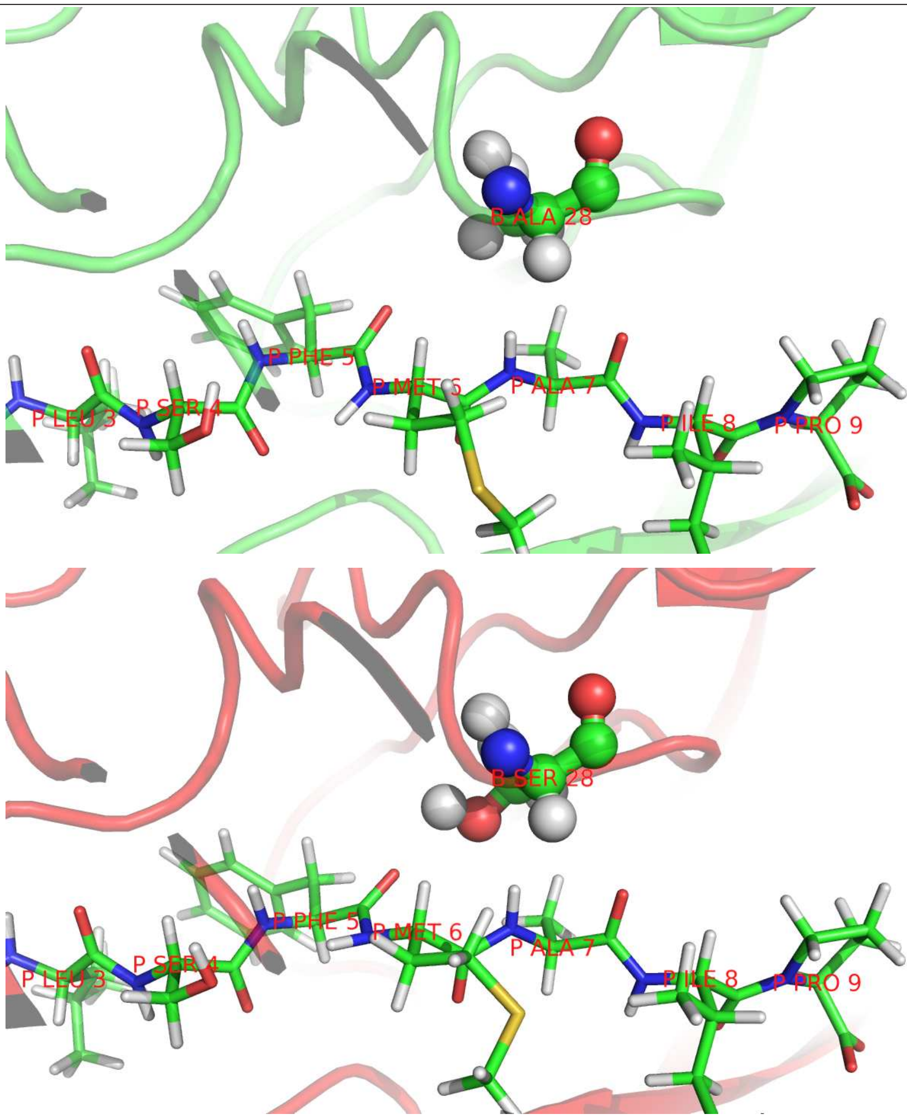

WT

M24

Figure S9. Chain B, residue 28.

D30T

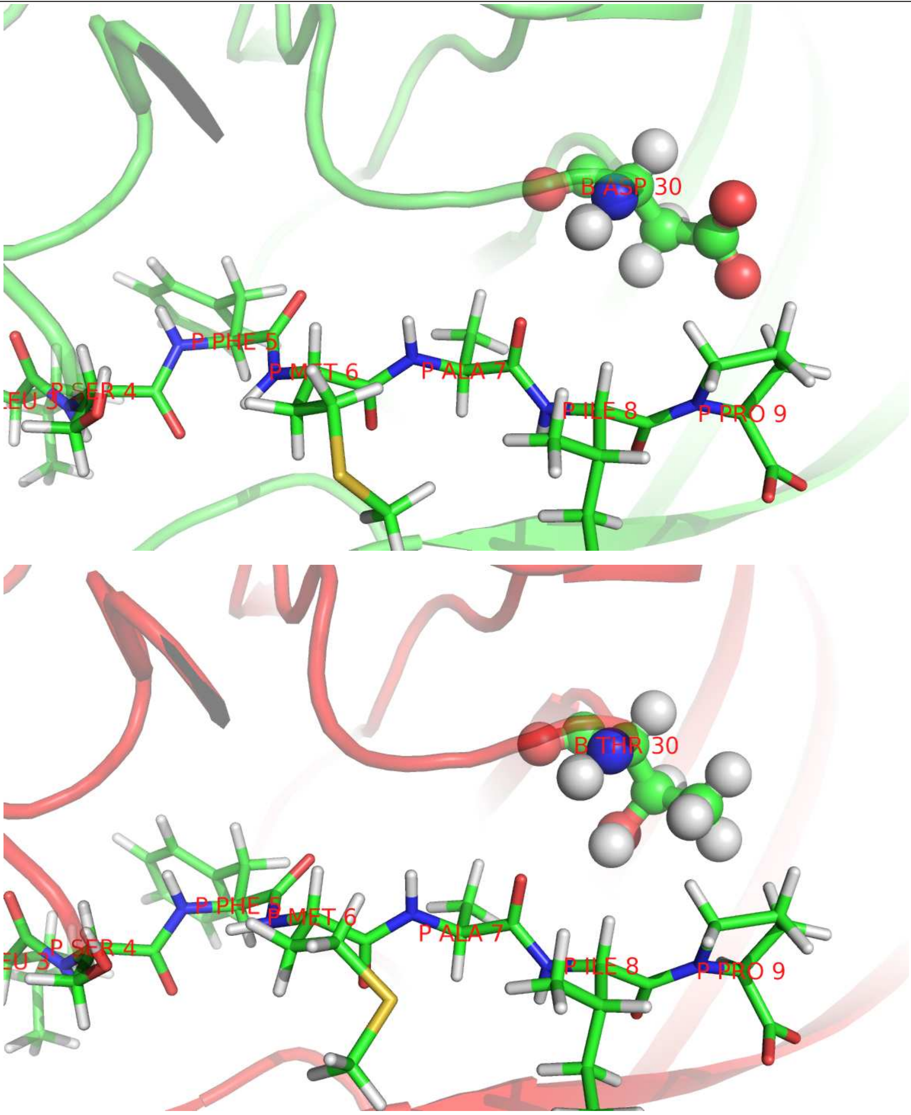

WT

M24

Figure S10. Chain B, residue 30.

K45A

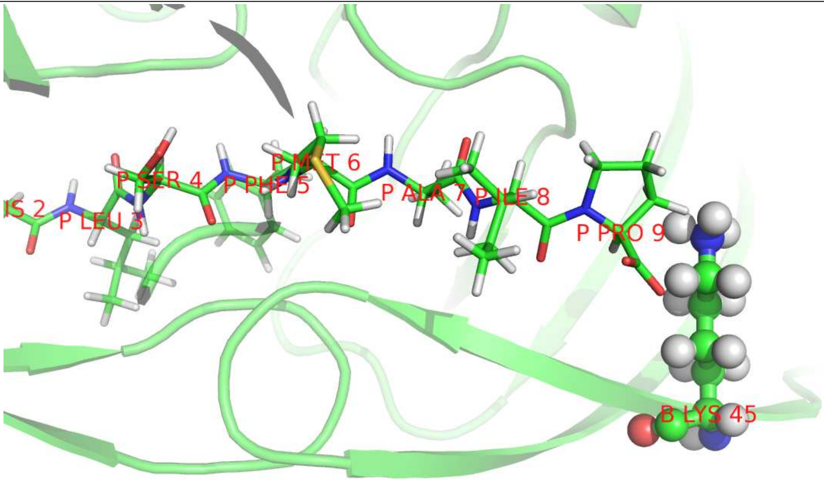

WT

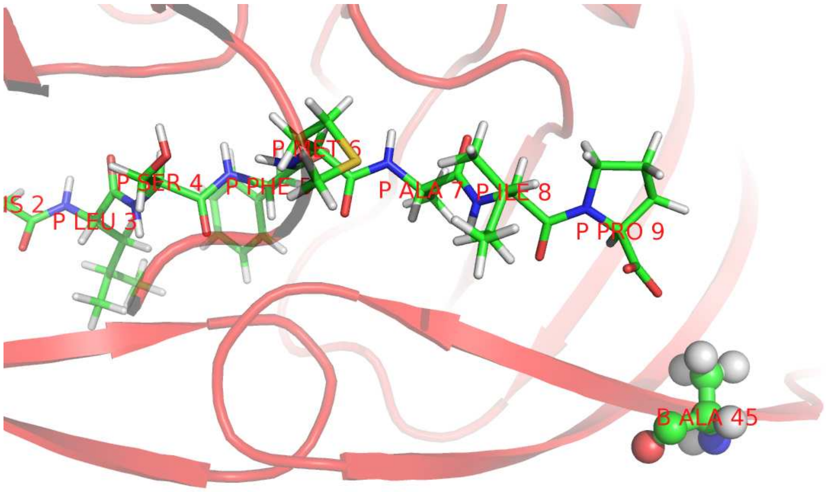

M24

Figure S11. Chain B, residue 45.

I50L

WT

M24

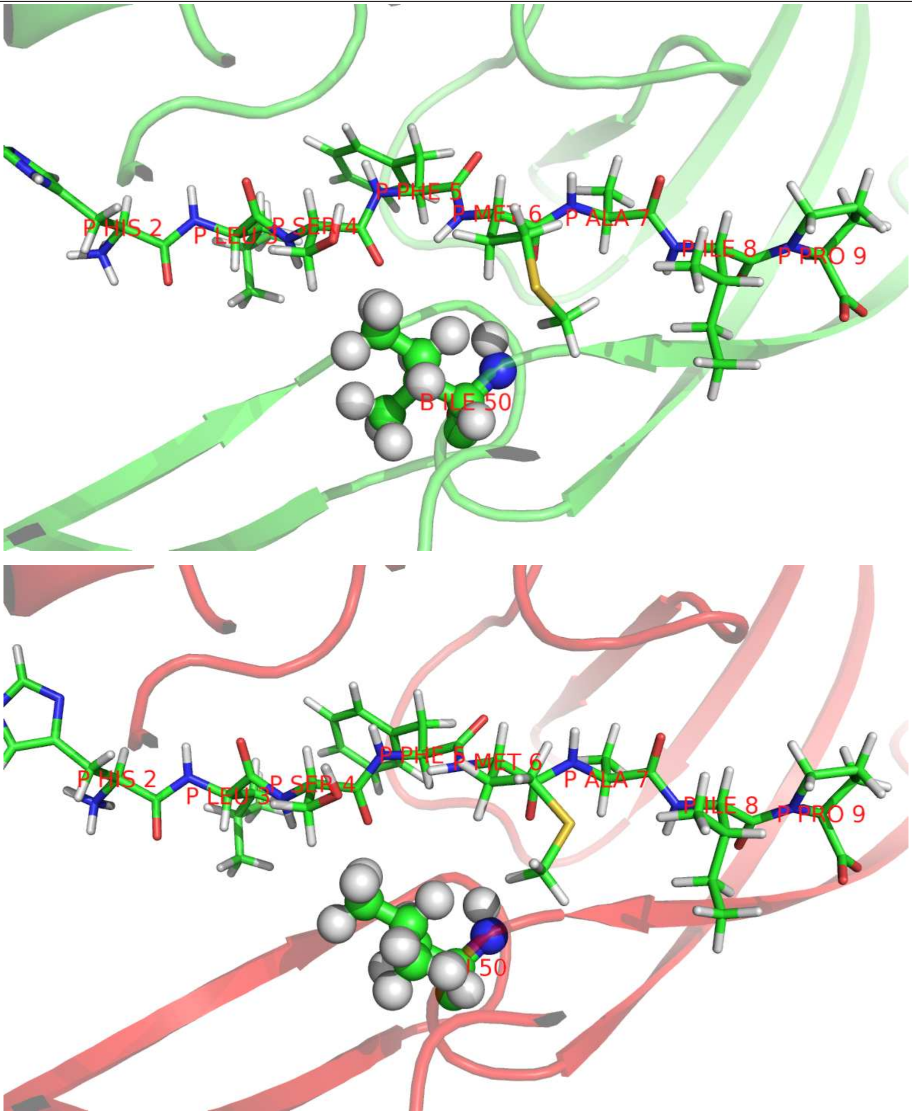

Figure S12. Chain B, residue 50.

P81L

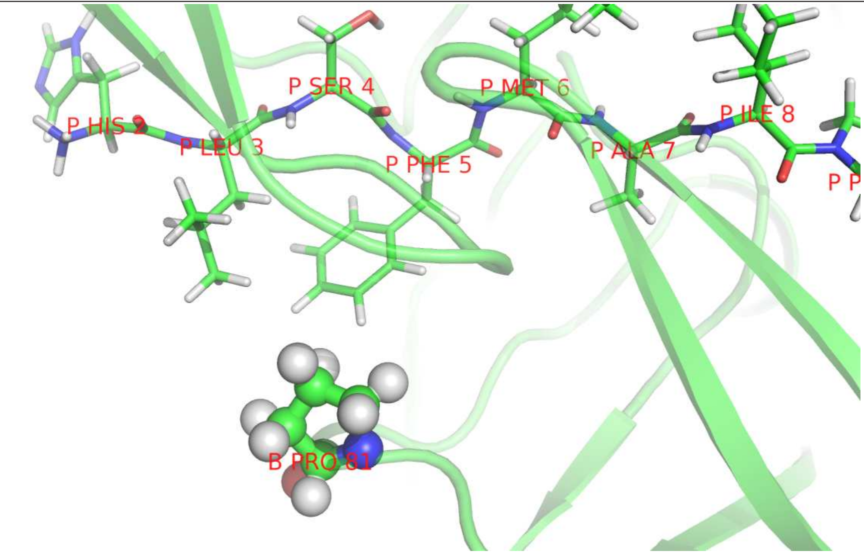

WT

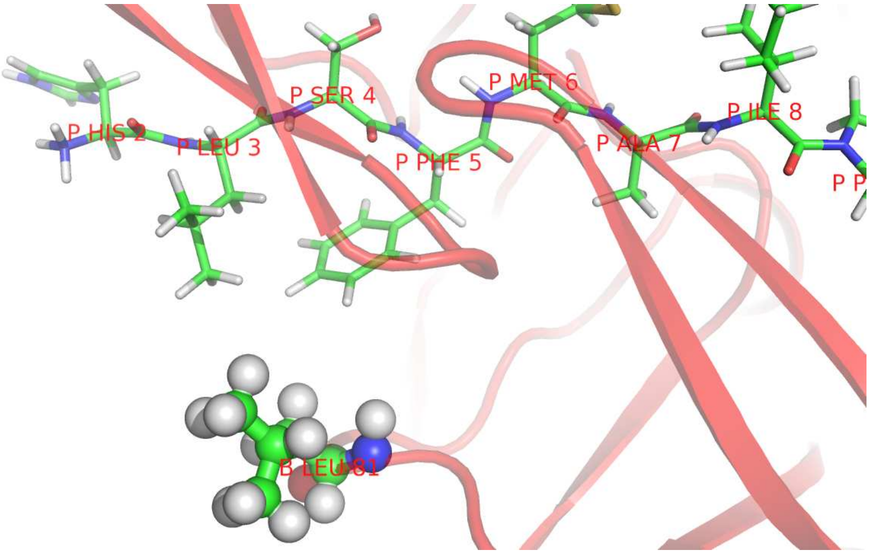

M24

Figure S13. Chain B, residue 81.

V82Y

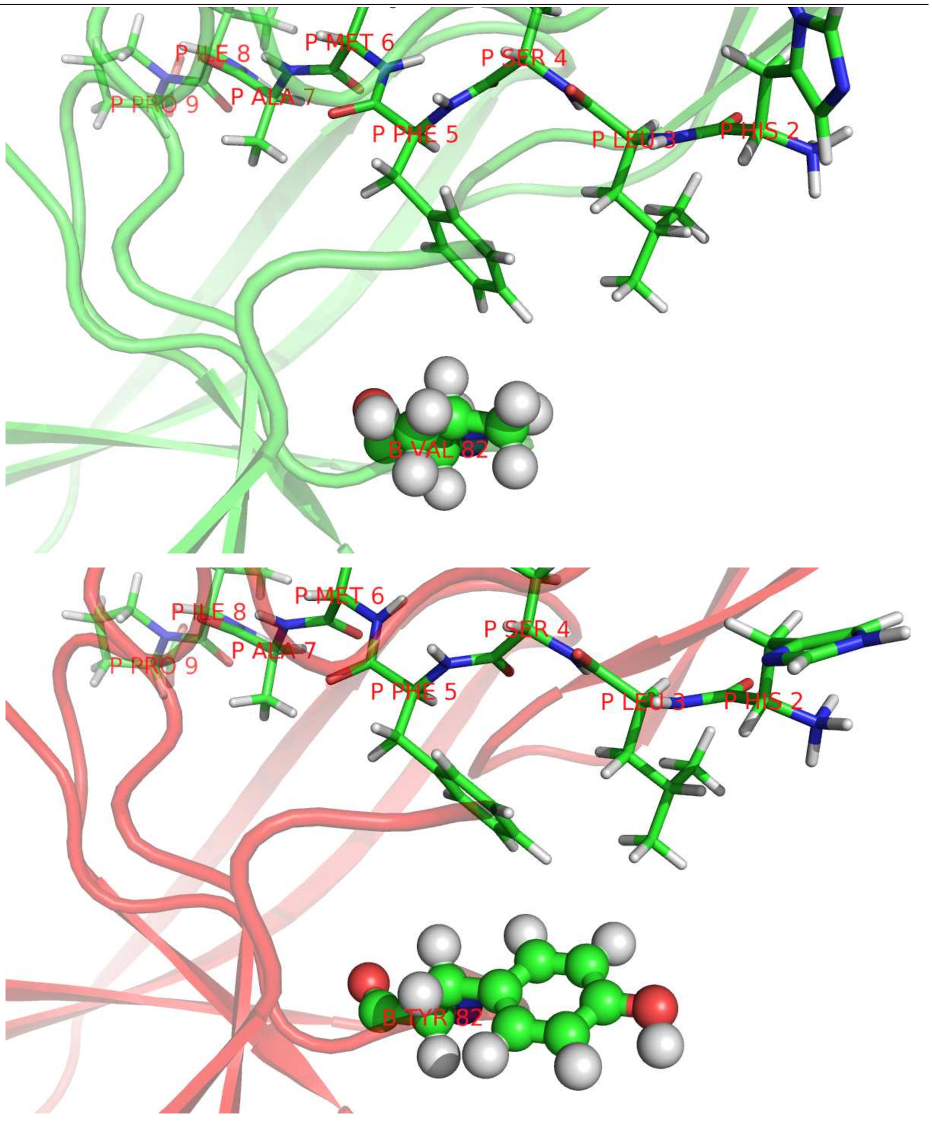

WT

M24

Figure S14. Chain B, residue 82.
